# β-Elemene enhances radiosensitivity in non-small-cell lung cancer by inhibiting epithelial–mesenchymal transition and cancer stem cell traits via Prx-1/NF-kB/iNOS signaling pathway

**DOI:** 10.18632/aging.202291

**Published:** 2020-12-09

**Authors:** Kun Zou, Zongjuan Li, Yang Zhang, Lin Mu, Miao Chen, Ruonan Wang, Wuguo Deng, Lijuan Zou, Jiwei Liu

**Affiliations:** 1 The First Affiliated Hospital, Institute of Cancer Stem Cell and, The Second Affiliated Hospital, Dalian Medical University, Dalian, China; 2 Sun Yat-sen University Cancer Center, State Key Laboratory of Oncology in South China, Collaborative Innovation Center of Cancer Medicine, Guangzhou, China; 3 Qingdao University Medical College Affiliated Yantai Yuhuangding Hospital, Yantai, China

**Keywords:** β-elmene, radiosensitivity, stem cell, Prx-1, EMT

## Abstract

Radiation therapy is widely used to treat a variety of malignant tumors, including non-small-cell lung cancer (NSCLC). However, ionizing radiation (IR) paradoxically promotes radioresistance, metastasis and recurrence by inducing epithelial-mesenchymal transition (EMT) and cancer stem cells (CSCs). Here, we developed two NSCLC radioresistant (RR) cell lines (A549-RR and H1299-RR) and characterized their motility, cell cycle distribution, DNA damage, and CSC production using migration/invasion assays, flow cytometry, comet assays, and sphere formation, respectively. We also evaluated their tumorigenicity *in vivo* using a mouse xenograft model. We found that invasion and spheroid formation by A549-RR and H1299-RR cells were increased as compared to their parental cells. Furthermore, as compared to radiation alone, the combination of β-elemene administration with radiation increased the radiosensitivity of A549 cells and reduced expression of EMT/CSC markers while inhibiting the Prx-1/NF-kB /iNOS signaling pathway. Our findings suggest that NSCLC radioresistance is associated with EMT, enhanced CSC phenotypes, and activation of the Prx-1/NF-kB/iNOS signaling pathway. They also suggest that combining β-elemene with radiation may be an effective means of overcoming radioresistance in NSCLC.

## INTRODUCTION

Lung cancer, the most common cause of cancer-related deaths, exhibits two major histological types, non-small-cell lung cancer (NSCLC) and small-cell lung cancer (SCLC), with the former being more prevalent (~80%–85% of all cases) than the latter. Unfortunately, NSCLC commonly presents metastases in distant organs [1, 2]. While radiotherapy can be used to treat patients with unresectable NSCLC, its effects remain unsatisfactory despite continuous developments in the field [3–5].

Many factors impact the response of tumor cells to radiotherapy. Ionizing radiation can activate some genes associated with apoptosis, DNA damage repair, cell adhesion and angiogenesis signaling pathways. These genes, in turn, may mediate cellular responses to radiation, influencing the effects of radiotherapy [6]. Recent studies show that epithelial-mesenchymal transition (EMT) and cancer stem cells (CSCs) promote radioresistance and lung cancer recurrence after radiotherapy [7, 8]. EMT promotes the disassembly of epithelial cell-junctions, the loss of epithelial polarity, and the formation of molecular assemblies allowing cell migration and invasion correlated with poor prognosis. EMT has been broadly studied in various types of tumors and is thought to contribute to radioresistance. Some studies suggest that EMT can be triggered by extracellular stimuli such as radiation [9, 10]. CSCs are a class of pluripotent cells uniquely capable of seeding new tumors, and the onset of EMT increases CSC subpopulations [11, 12]. Thus, determining the molecular mechanisms underlying radiation-induced increases in EMT and CSC could lead to improved therapeutics for NSCLC.

β-Elemene, the active component of elemene, is extracted from the Chinese medicinal herb Curcuma Wenyujin, which exerts anti-tumor effects in a broad range of solid tumors. In China, β-elemene has been applied in clinical practice, and it causes fewer side effects than other commonly-used therapeutic agents that are cytotoxic [13–15]. Previously, we showed that β-elemene increased the sensitivity of lung cancer cells to radiation and that Prx-1 might be the major target for radiosensitization [16, 17]. Here, we investigated whether EMT and CSCs promoted NSCLC radiation resistance, the underlying molecular mechanisms, and the use of β-elemene in combination with RT to treat NSCLC.

## RESULTS

### Establishment and validation of A549-RR and H1299-RR cells

**Figure 1:**
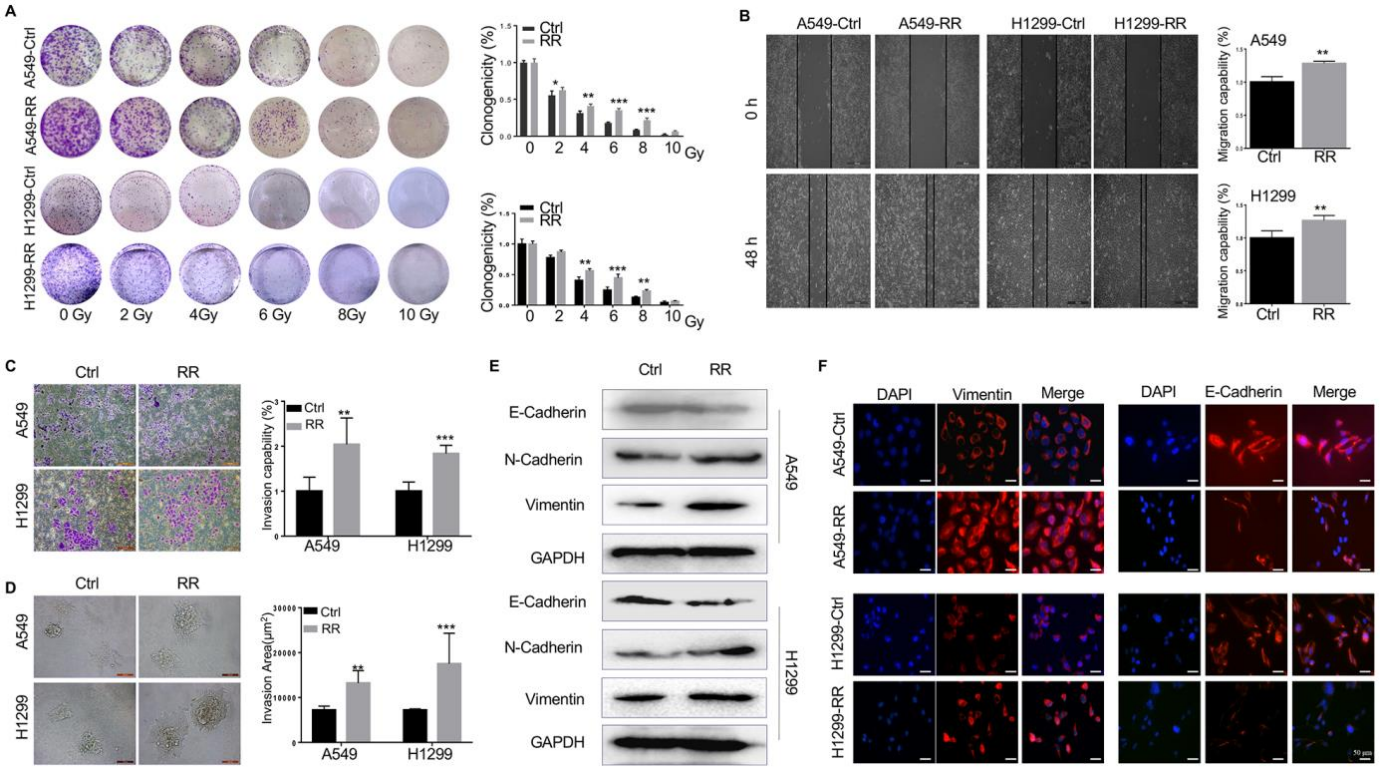
**Different radiosensitivity, motility and EMT potential in radioresistant and paternal cells.** (**A**) Count of cell colonies formed 10-12 days after 2-10 Gy radiation treatment. (**B**) Cell migration analyzed by wound healing assay using wound gap photographs for A549-RR, A549-control, H1299-RR and H1299-control cells. **P < 0.01, significant difference between radioresistance and control cells. (**C**) A549-RR, A549-control, H1299-RR and H1299-control cells were subjected to Matrigel invasion assay and photographed (magnification 20×, scale bar 100 μm) for the analysis of their invasion capacity. **P < 0.01, significant difference between A549-RR and A549-control cells. (**D**) Representative 3D-invasion images of cells. Scale bar:100 μm. (**E**) Protein levels of E-Cadherin, N-Cadherin and Vimentin measured by Western blot. GAPDH served as the loading control. (**F**) Vimentin (red) immunofluorescence images of cells. Nuclei were stained with PI (blue) All data from the RR cell line were collected between 5 and 6 weeks post-radiation treatment. All results were from three independent experiments and are presented as mean ± SD. *P*-values were calculated by student’s *t*-test.

To generate A549-RR and H1299-RR cell lines, A549 and H1299 cells were treated with different doses of radiation (2, 4, 6 and 8 Gy) for five consecutive days. Following the final radiation, A549 and H1299 cells were maintained in a humidified incubator for 35 days for recovery. The results indicated that 2 Gy/day for five consecutive days was the maximum tolerance dose. Next, both A549, A549-RR, H1299 and H1299-RR cells were exposed to a range of single radiation doses (2-10 Gy) to test their radioresistance. We found that the clonogenicity of A549, A549-RR, H1299 and H1299-RR cells was inhibited in a dose-dependent manner, but the surviving fraction in A549-RR and H1299-RR cells were much larger than that in A549 and H1299 cells as the radiation dose increased (Figure 1A).

### Motility induction and acquisition of EMT of A549-RR and H1299-RR cells

We used wound-healing and transwell assays to assess the migration and invasion ability of A549-RR and H1299-RR cells. As shown in Figure 1B, 1C, A549-RR and H1299-RR cells had higher migration and invasion capacity than the parental cells of A549 and H1299. Furthermore, in 3D spheroid basement membrane invasion assays, the results showed that A549-RR and H1299-RR cells have more invasion areas than the parental cells, cell spheroids and greatly increased the number of invading cell protrusions, consistent with an increased invasion phenotype (Figure 1D) The dramatic increase in invasion of A549-RR and H1299-RR cells suggested that radioresistance might enhance their invasion capacity. Since EMT promotes tumor invasion and metastasis in various malignancies, including NSCLC, we then measured the levels of EMT-related proteins. Western blot and immunofluorescence showed that the epithelial marker E-cadherin was downregulated while the mesenchymal markers N-cadherin and Vimentin were upregulated in A549-RR and H1299-RR cells (Figure 1E, 1F).

### Increased CSC phenotypes in A549-RR and H1299-RR

**Figure 2:**
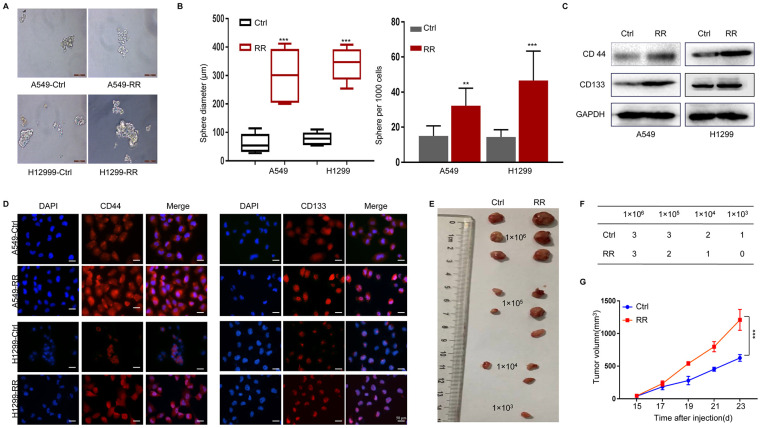
**Enhanced CSC properties in A549-RR and H1299-RR cells.** (**A**) Radioresistant and control cells were grown in an ultra-low attachment plate as indicated for 14 days. Representative images of tumor cell spheres were taken for quantification. (**B**) The diameter and number of tumor cell spheres in (**A**). (**C**) CD44 and CD133 protein levels in cell lysates measured by Western blot. (**D**) Immunofluorescence images of CD44 and CD133 (red) in radioresistant and control cells. Nuclei are stained with PI (blue). (**E**) Representative images of the xenografts. (**F**) Tumor incidence in xenograft of A549-RR and A549-control cells. (**G**) Tumor volume was measured once every 2 days and was calculated as: V = (width^2^×length)/2. All data used in RR cell lines were based on cells between 5 and 6 weeks post radiation treatment. All results were from three independent experiments, and the data are shown as mean ± SD. *P*-values were calculated by student’s *t*-test.

CSCs possess self-renewal capacity and can persistently proliferate to initiate tumors upon serial transplantation. Growth as tumor spheres is considered to be a surrogate marker for CSC and self-renewal ability in epithelial cancers [19]. We analyzed the tumor sphere formation in both A549, A549-RR, H1299 and H1299-RR cells. As shown in Figure 2A, 2B, sphere numbers and sizes were increased in A549-RR and H1299-RR cells. Next, we examined the expression of CD44 and CD133 by Western blot and found that A549-RR and H1299-RR cells demonstrated enhanced expression of these CSC markers compared with A549 and H1299 cells (Figure 2C) Similar results were observed for the CD44 and CD133 levels by immunofluorescence staining, which were increased in A549-RR and H1299-RR cells (Figure 2D) To analyze the tumorigenicity of the radioresistant cells that exhibited CSC-like properties, we then injected A549 and A549-RR cells as 10-fold dilution series from 1x10^6^ to 1x10^3^ into subcutaneous sites of nude mice. As shown in Figure 2E, 2F, all the mice that were injected with 1 x 10^6^ and 1 x 10^5^ A549-RR cells formed tumors, whereas no masses occurred when an equal number of A549 cells were injected. Moreover, the mice with subcutaneous xenografts of A549-RR similarly displayed remarkable tumor growth enhancement (Figure 2G) Our findings suggested that RR cells had increased CSC phenotypes.

### Redistribution of cell cycles and attenuated DNA damage response in A549-RR cells

**Figure 3:**
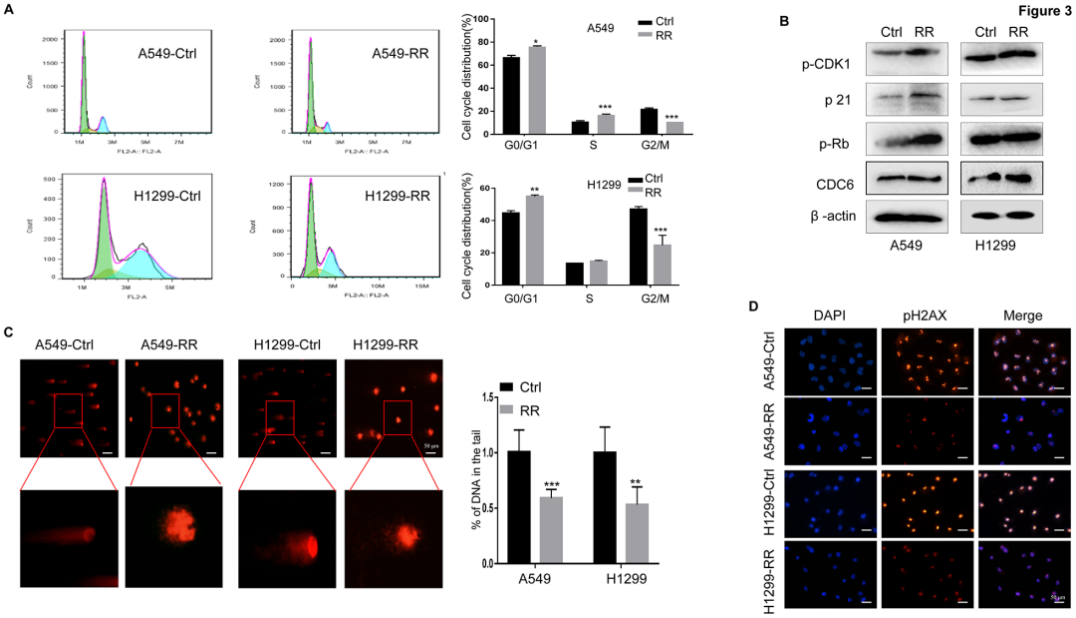
**A549-RR and H1299-RR cells showed cell cycle redistribution and reduced DNA damage response.** (**A**) Cell cycle distribution was analyzed with flow cytometry. The percentage of cells at each phase of the cell cycle was quantified. (**B**) Cell cycle-related protein (p-CDK1, p21, p-Rb and CDC6) levels were measured using Western blot. (**C**) A549-RR, A549-control, H1299-RR and H1299-control cells were exposed to radiation at the same dose of 4 Gy and collected for comet assay as described in materials and methods. The percentage of DNA in the tail was calculated for 50 random cells. **P < 0.01, significant difference between radioresistant and control cells. (**D**) γH2AX, the indicator for DSBs, was detected by immunofluorescence staining. The results in (**A**) and (**C**) are the mean ± SEM of at least three independent experiments.

Cells in different phases of the cell cycle exhibit different radiation sensitivity. In general, cells are most sensitive to radiation during the G2/M phase, less sensitive during G1 phase, and least sensitive near the end of the S phase [20, 21]. In our present study, the percentage of G0/G1 and S cell was increased, whereas the percentage of G2/M cell was obviously reduced in A549-RR and H1299-RR cells compared with A549 and H1299 control cells (Figure 3A) Consistent with the cell cycle distribution, the important cell cycle checkpoint proteins p-CDK1, p21 and p-Rb proteins was increased in A549-RR and H1299-RR cells (Figure 3B) Cell division cycle 6 (CDC6) is an essential regulator of DNA replication and cell cycle arrest that is required for Chk1 activation and could be induced by the upregulation of p21 [22]. CDC6 overexpression during G2 phase blocks mitotic entry by activating Chk1 [23]. In our study, we found that the radioresistant lung cells tend to have a higher expression of CDC6 (Figure 3B) The results suggested that resistance to radiation might be, at least in part, promoted by the redistribution of cell cycle phases that increased the proportion of cells in the least sensitive stage.

The DNA double-strand is highly susceptible to radiation damage, which can directly ionize DNA or indirectly stimulate reactive oxygen species (ROS) production, both resulting in DNA damage. DNA double-strand breaks (DSBs) are the most lethal form of DNA damage for tumor cells [24, 25]. To examine the DNA damage response in both A549, A549-RR, H1299 and H1299-RR cells, the same therapeutic dose of 4 Gy radiation was performed and compared. In the comet assay, we found that the A549 and H1299 control cells had longer trailing after 4 Gy radiation, which indicated more DSBs (Figure 3C) To further assess the DNA damage response, we examined phosphorylation of histone 2AX on serine 139 (γH2AX), an indicator for DSBs, by immunofluorescence staining [26, 27]. For A549 and H1299 control cells, 4 Gy radiation treatment led to increased numbers of γH2AX foci when compared to A549-RR and H1299-RR cells (Figure 3D) These results suggested that A549-RR and H1299-RR cells were more resistant to DSBs caused by IR.

### β-Elemene inhibited EMT and CSC phenotypes induced by radiotherapy

**Figure 4:**
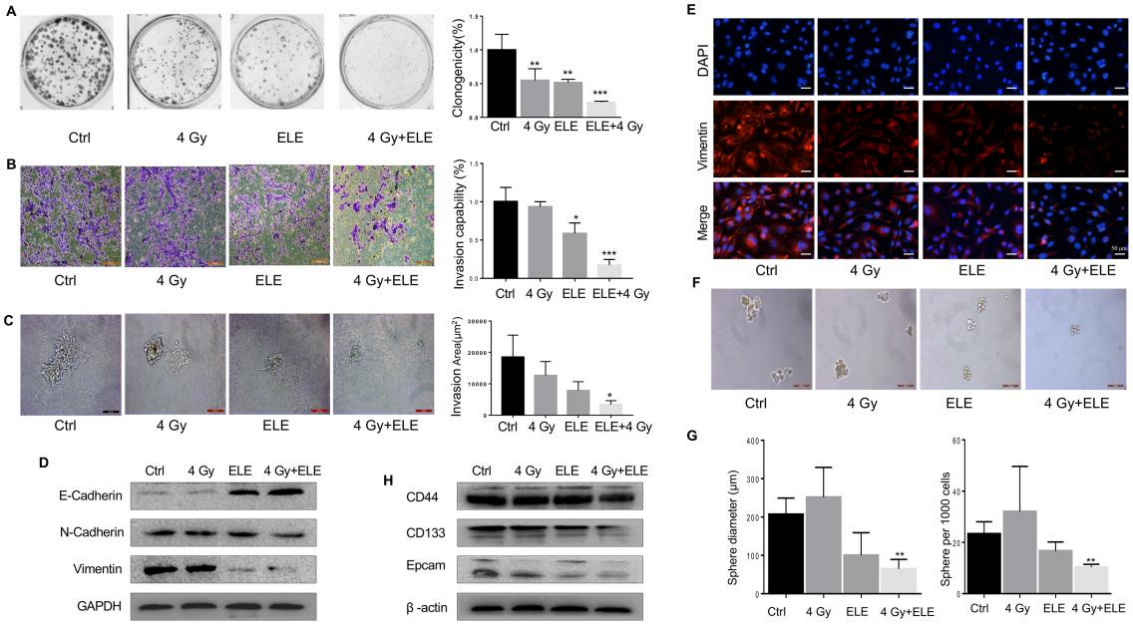
**Effect of β-elemene and RT on the expression of EMT/CSC markers and radiosensitivity in A549 cells.** A549 cells were treated with single RT (4 Gy) for 12 h or β-elemene 12 h prior to RT (4 Gy). (**A**) Representative images of colony formation for the different treatments. (**B**) After corresponding treatment, the cells were subjected to Matrigel invasion assay and photographed. Invasion capability of cells was calculation. (**C**) Representative 3D-invasion images of cells. Scale bar:100 μm. (**D**) Protein levels of Vimentin, E-Cadherin and N-Cadherin were measured by Western blot. (**E**) Immunofluorescence images of Vimentin (red) Nuclei were stained with PI (blue). (**F**) Representative images of spheroid formation after the different treatments. (**G**) The diameter and number of tumor cell spheres in (**E**). (**H**) Protein levels of CD133, CD44, and Epcam measured by Western blot.

β-Elemene is an extract of traditional Chinese medicine, which is widely used in clinical treatment and causes fewer side effects than other cytotoxic agents [13–15]. Radiation alone and combination treatment with β-elemene and radiation were next performed and compared. After combination treatment by β-elemene and 4 Gy irradiation, A549 cells showed a large reduction in colony formation, when compared with the cells treated with radiation alone (Figure 4A) To determine the inhibition effect of β-elemene on invasion, transwell assay was performed in A549 cells. As shown in Figure 4B, combination treatment with β-elemene and radiation inhibited cell invasion. We next performed 3D invasion assay and found that the invasion area of A549 was decreased in the combined treatment group (Figure 4C) To further confirm the effect of β-elemene, we checked the levels of the key proteins involved in EMT. Western blot and immunofluorescence results indicated that combination treatment could block the EMT process induced by radiation, upregulating the epithelial marker E-cadherin and downregulating of N-cadherin and Vimentin (Figure 4D, 4E).

Next, we tested whether the combination treatment with β-elemene and radiation could influence CSC and found that it dramatically decreased sphere numbers and sizes (Figure 4F, 4G) Sphere formation efficiency (SFE) of cells treated with both agents was ~31% lower compared with radiation alone. Next, we measured the expression of several recognized CSC markers including CD133, CD44, and Epcam. Consistent with the results above, β-elemene could block the expression of those CSC markers (Figure 4H) In conclusion, combination treatment with β-elemene and RT reversed EMT expression and reduced the levels of CSC marker expression in A549 cells compared with RT alone.

### β-Elemene reversed the cell cycle redistribution induced by radiation and inhibited DNA damage repair

**Figure 5:**
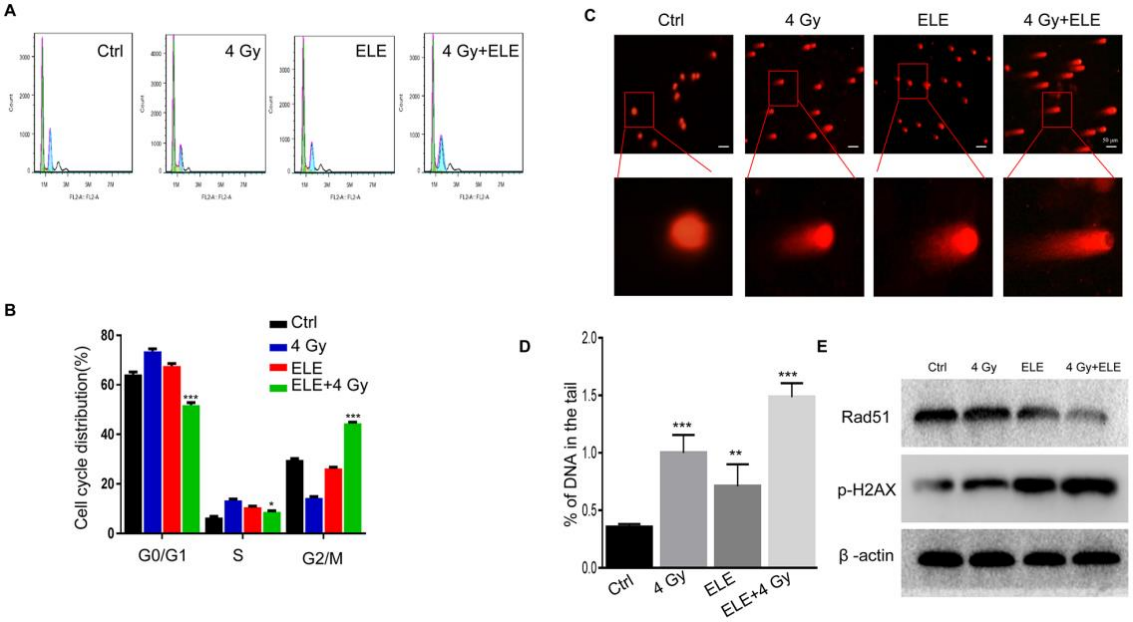
**Effect of β-elemene and RT on cell cycle and DNA damage repair in A549 cells.** A549 cells were treated with single RT (4 Gy) or β-elemene prior to RT (4 Gy). (**A**) After each treatment, cell cycle distribution was analyzed with flow cytometry. (**B**) The percentage of cells at each phase of the cell cycle was quantified. (**C**) A549 cells were harvested 48 h after different treatments and subjected to the comet assay, as described. (**D**) The percentage of DNA in the tail was calculated for 50 random cells. (**E**) DSB marker (γH2AX), and homologous recombination pathway-related proteins (RAD51) were measured by Western blot. Representative images from three independent experiments are shown.

The therapeutic effects of IR are traditionally associated with changes in cell cycle distribution and DNA damage repair capacity. We examined whether β-elemene could reverse the influence of radiotherapy on the cell cycle and DNA damage. Combination treatment with β-elemene and radiation reduced the proportion of cells in G0/G1 and S phases and dramatically increased the proportion of cells in G2/M phase when compared with radiation alone in A549 cells (Figure 5A, 5B) Next, we evaluated DNA damage through comet assay and found that the combination treatment group had longer trailing after 4 Gy irradiation, indicating more DNA damage (Figure 5C, 5D) Then, we measured the expression of γ-H2AX (double-strand break marker) and Rad51 (double-strand break repair protein), and found that the expression of γ-H2AX was increased, whereas the expression of Rad51 was reduced in the combination treatment group compared with radiation alone in A549 cells (Figure 5E) These results suggested that β-elemene could increase the radiosensitivity of A549 cells by regulating the cell cycle and inhibiting DNA damage repair.

### β-Elemene sensitized A549 cells to radiation through the Prx-1/NF-kB/iNOS pathway

**Figure 6:**
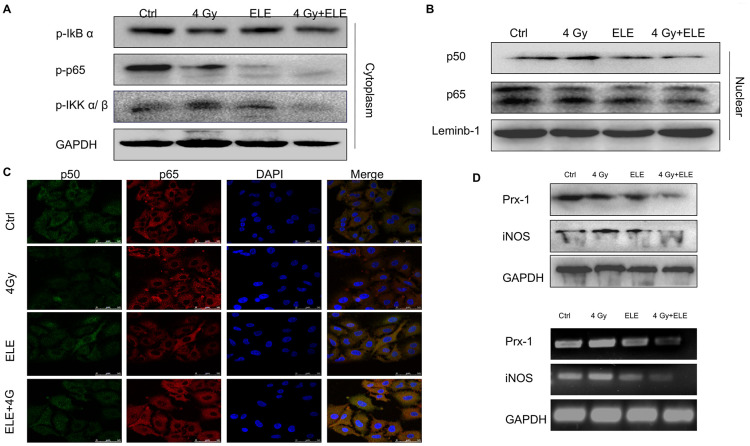
**The effect of β-elemene and RT on the expression of Prx-1/NF-kB /iNOS pathway.** A549 cells were treated with single RT (4 Gy) or β-elemene prior to RT (4 Gy) Cell lysates were extracted 12 h after RT. (**A**) The expression of p-IkBα, p-IKK α/β, and p-p65 in cytoplasm were measured by Western blot. (**B**) The cytoplasmic and nuclear proteins were separated, and the expression of p50/p65 in the nucleus was measured by Western blot. (**C**) The subcellular localization of p50, p65 in A549 cells treated with β-elemene or RT alone or their combination was examined by confocal microscopy. More than 100 cells were inspected per experiment, and the cells with typical morphology were presented. (**D**) The A549 cells were treated with β-elemene or RT alone or their combination. The expression of Prx-1 and iNOS at mRNA and protein levels were measured by RT-RCR and Western blot.

Nuclear factor-κB (NF-κB) is part of the early response of tumor cells to radiation and triggers cellular defense mechanisms [28]. Studies have shown that disruption of NF-κB signaling affects the migration, development and radiosensitivity of tumors [29, 30]. Therefore, we hypothesized that β-elemene might exert its effect via the NF-κB signaling pathway. To test this hypothesis, we treated A549 cells with β-elemene, irradiation or the two in combination. After 48 hours, cytoplasmic and nuclear proteins were separated and the expression of key proteins involved in NF-κB signaling pathway was measured. The results demonstrated that β-elemene combined with irradiation markedly decreased the phosphorylation of IκBα, iKKα/β and p65 in the cytoplasm compared with irradiation alone (Figure 6A) Furthermore, the expressions of p50 and p65 in the nucleus were decreased when treated with β-elemene and irradiation (Figure 6B) We performed immunofluorescence assay to further observe the localization of NF-κB. We found that irradiation alone promoted the translocation of NF-κB p50/p65 from the cytoplasm to nuclei, whereas β-elemene blocked the translocation of NF-κB p50/p65 induced by irradiation (Figure 6C) The inducible nitric oxide synthase (iNOS) promoter contains specific binding sites for NF-κB [31, 32]; thus, we studied the effect of β-elemene on iNOS and found that the transcription and expression of iNOS was decreased when cells were treated with β-elemene and irradiation. In contrast, treatment with irradiation alone increased the expression of iNOS at the mRNA and protein levels (Figure 6D).

Peroxiredoxins (Prxs) were proposed to function as damage-associated molecular patterns (DAMPs), and Prx-1 increases the expression of iNOS and the nuclear translocation of NF-κB p65 [33, 34]. Our previous study also found that Prx-1 might be a potential target for radiosensitization of elemene. As is shown in Figure 6D, a combination of β-elemene and irradiation treatments markedly inhibited the transcription and expression of Prx-1, suggesting that β-elemene exerted radiosensitization effects through the Prx-1/NF-kB /iNOS pathway.

### β-Elemene and radiation synergistically inhibited tumor growth in xenograft mouse models

**Figure 7:**
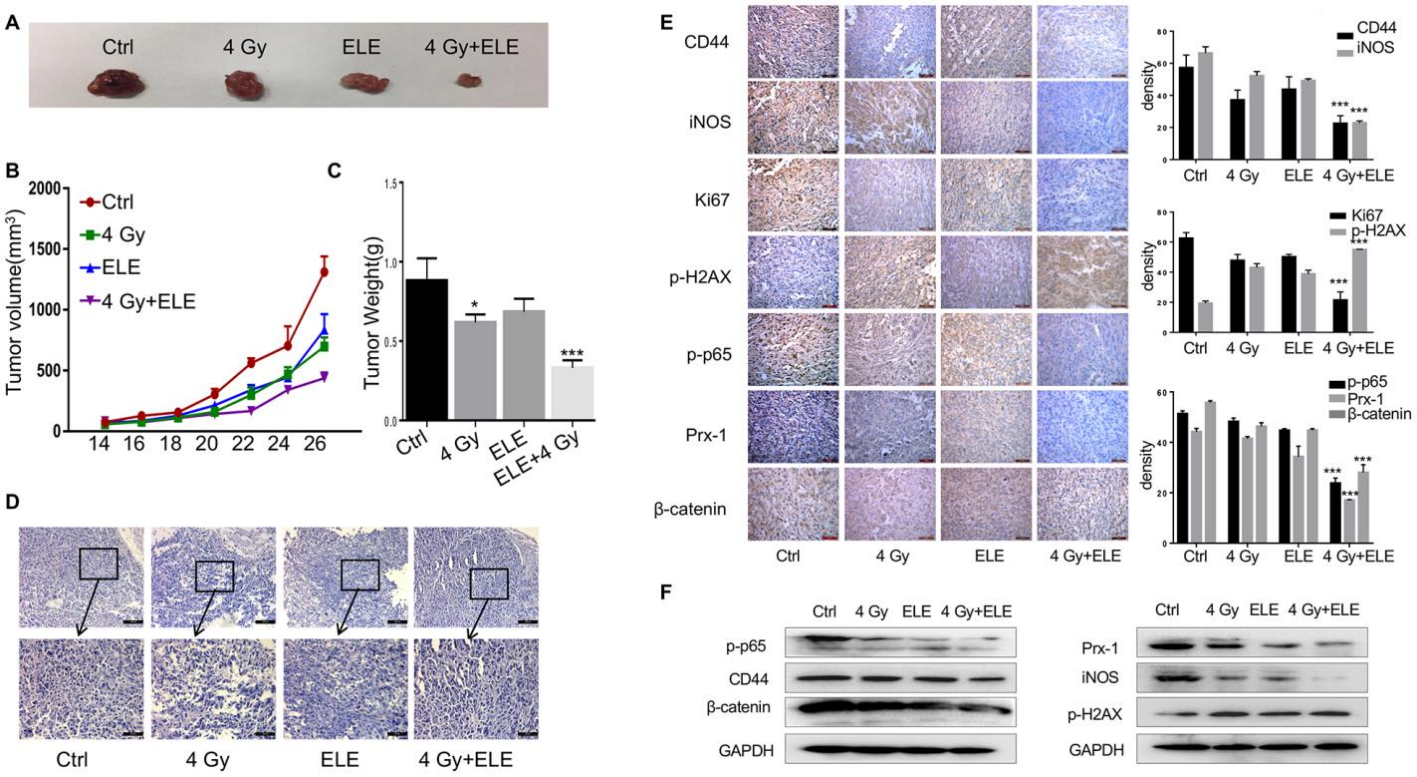
**The effect of β-elemene and RT on tumor growth in a xenograft mouse model of human non-small-cell lung cancer (NSCLC).** Female athymic nude mice aged 4-5 weeks old were used in the study. A549 cells (5×10^6^ in 100 μL PBS) were injected subcutaneously into the left flank of each mouse. The experiment was performed as described in materials and methods. The xenografts were harvested after two weeks. (**A**) Representative images of the xenografts. (**B**) Tumor volume was measured once every 2 days and was calculated as: V = (width^2^×length)/2. (**C**) Tumor weight. (**D**) Representative images of H&E staining. (**E**) Immunohistochemical analysis of Ki67, CD44, iNOS, β-cadherin, p-H2AX, p-65 and Prx-1 protein expression in tumor samples. (**F**) Protein level of CD44, iNOS, β-cadherin, p-H2AX, p-65 and Prx-1.

We also evaluated the effect of combination treatment with β-elemene and radiation on tumor growth *in vivo* in immunodeficient BALB/c mice. A549 cells were injected subcutaneously into nude mice, and the mice were divided into four groups after eight days. Tumor volume was measured every two days until the mice were sacrificed. The combination therapy dramatically suppressed NSCLC tumor growth as indicated by reductions in size, volume and weight compared to the control and single-agent groups (Figure 7A–7C) In addition, H&E staining showed that the β-elemene and irradiation co-treated tumor cells had larger and more deformed nuclei with high nucleocytoplasmic ratio (Figure 7D) Consistently, Ki67 staining displayed that cells co-treated with β-elemene and irradiation had a remarkably decreased proliferation index (Figure 7E) Consistent with our *in vivo* results, immunohistochemical staining assay showed that CD44, β-catenin and γ-H2AX were expressed at lower levels in the combination-treatment group (Figure 7E) To explore the inhibition of signaling pathways by combination treatment, we further analyzed the expression of the Prx-1/NF-kB/iNOS signaling pathway in the xenograft tumors and founded that Prx-1, iNOS and p-p65 were markedly reduced (Figure 7E) Furthermore, Western blot of the cell lysates from the xenograft tumors confirmed such changes in the expression of these proteins (Figure 7F) Our results showed that β-elemene and irradiation synergistically exerted antitumor effects in NSCLC and inhibited the Prx-1/NF-kB /iNOS signaling pathway.

## DISCUSSION

Irradiation is a major therapeutic tool for NSCLC treatment. However, irradiation paradoxically enhances the migration and invasiveness of cancer cells by inducing EMT and cancer stem cell (CSC) phenotypes that promote radioresistance, metastasis and recurrence [35]. Nonetheless, the underlying molecular mechanisms remain unclear and their identification could help to find therapeutic targets and develop more effective treatments to overcome radioresistance and recurrence after RT in NSCLC. Here, we developed novel A549-RR and H1299-RR cell lines derived from clones that had survived after irradiation, thereby mimicking clinical RR and metastasis after RT.

Emerging evidence suggests that epithelial–mesenchymal transition (EMT) and cancer stem cells (CSCs) promote cancer radiation resistance. EMT is characterized by the loss of adhesion, negative expression of E-cadherin, and the acquisition of mesenchymal characteristics, such as expression of vimentin. CSCs may produce tumors through self-renewal and differentiation into multiple cell types [36, 37]. These CSCs can provide a reservoir of cells that cause tumor recurrence even after therapy. Here, we found that A549-RR and H1299-RR cells tended to have stronger EMT and CSC phenotypes compared with those in A549 and H1299 control cells. Furthermore, combination treatment with β-elemene and radiation inhibited the EMT and CSC transdifferentiation induced by radiation, suggesting that β-elemene could be used as an effective treatment against radioresistance in NSCLC.

The different responses of cancer cells to radiation are largely determined by cell cycle distribution and DNA repair capacity [38]. Here, we found that the G2/M phase was decreased in A549-RR and H1299-RR cells while cell cycle regulators p-Rb, p-CDK1, CDC6 and p21 were increased. CDC6 is an androgen receptor (AR) target gene that regulates DNA replication and checkpoint mechanisms. CDC6 is required for Chk1 activation, and overexpression of CDC6 during G2 phase blocks mitosis by activating Chk1, which inhibits G2/M progression [23, 39, 40]. Moreover, CDC6 inhibits E-cadherin expression, and overexpression of CDC6 can promote EMT and tumor development [41]. In our study, we found that A549 -RR and H1299-RR cells had a higher expression of CDC6. The function of CDC6 is complex, and the specific mechanisms by which it affects radiosensitivity in lung cancer needs further study.

Radiation kills cells by causing DNA damage, with DSB being the most dangerous type. γH2AX is highly specific and sensitive for monitoring both DSB initiation and resolution. Here, we found that A549-RR and H1299-RR cells had a shorter DNA trailing and lower expression of γH2AX compared with A549 and H1299 control cells when exposed to the same radiation dose. When treated with β-elemene and radiation, A549 cells showed a redistribution of G2/M phase, enhanced expression of γH2AX and reduced expression of Rad51, restoring the sensitivity to radiation. Our results showed that cell cycle and HR repair pathways might be involved in the radiosensitization induced by β-elemene and radiation combination treatment in A549 cells.

Nuclear factor-κB (NF-κB) expression is part of the early response of mammalian cells to ionizing radiation and triggers cellular defense mechanisms. In the absence of stimulation, NF-κB is sequestered in the cytoplasm but after exposure to IR, proteasomal degradation of IκB following phosphorylation by IKK leads to aberrant NF-κB activation and nuclear translocation. Additionally, the activity of the NF-κB transcription factor family is essential for EMT induction and maintenance. Thus, we further examined the synergistic effect of β-elemene and radiation on the NF-κB pathway. Our results showed that β-elemene and radiation synergistically blocked the translocation of NF-κB p50/p65 from the cytoplasm to nuclei and downregulated the transcription and expression of iNOS. Prx-1 exerts DNA-damage-associated functions, increasing the production of proinflammatory mediators, including nitric oxide (NO) metabolites, tumor necrosis factor-α (TNF-α), and interleukin-6 (IL-6), and is also induced by radiation in various types of cancer cells *in vitro* [42, 43]. Our team previously found that Prx-1 could be a potential target for the radiosensitization induced by β-elemene, but the underlying regulatory mechanisms remained unknown. Several studies have shown that Prx1 may directly activate the TLR4/NF-κB /iNOS signaling pathway and trigger inflammatory responses in macrophages [44, 45]. Here, we found that combination treatment with β-elemene and irradiation markedly inhibited the transcription and expression of Prx-1, suggesting that β-elemene exerted antitumor effects through the Prx-1/NF-kB /iNOS pathway.

**Figure 8:**
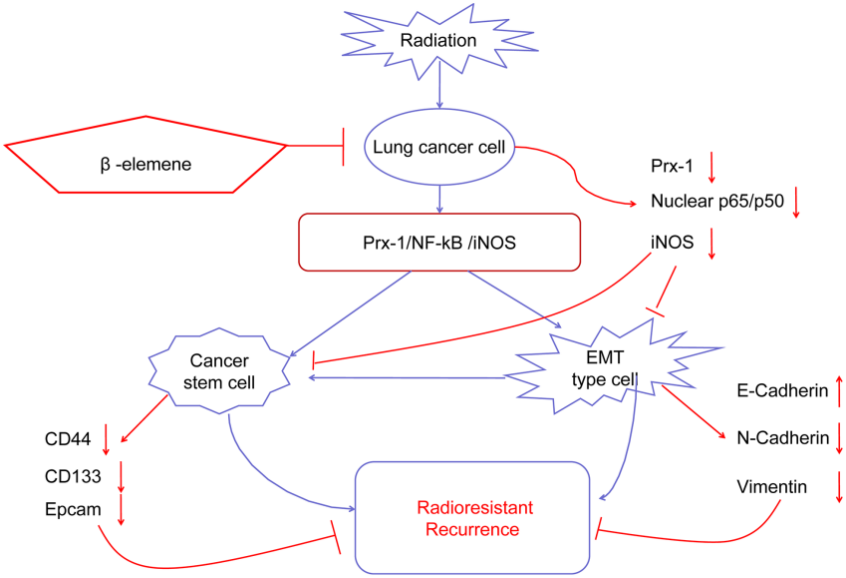
**Working model of β-elemene and RT on tumor growth in NSCLC cells.**

In summary, we demonstrate that NSCLC radioresistance is promoted by several mechanisms including EMT, CSCs, DNA damage, and abnormal cell cycle distribution, which result in cancer cell growth, survival, invasion, DNA repair and metastasis. β-elemene treatment combined with radiation can overcome NSCLC radioresistance and reverse the EMT and CSC transdifferentiation induced by radiation via the Prx-1/NF-kB/iNOS pathway (Figure 8) Our results highlight the combination of β-elemene with radiation as a potentially-promising treatment for NSCLC.

## MATERIALS AND METHODS

### Cell lines and cell culture

A549 and H1299 cells were obtained from American Type Culture Collection (ATCC) and grown in Dulbecco’s Modified Eagle’s Medium (DMEM) and 1640 Medium respectively supplemented with 10% fetal bovine serum (FBS) Cells were maintained in a humidified incubator with 5% CO_2_ at 37° C.

### Reagents and antibodies

β-Elemene was obtained from the National Institutes for Food and Drug Control (NIFDC, Beijing, China) Antibodies against β-actin, GAPDH, CD133, CD44, Prx-1, γ-H2AX, RAD51, CDC6 and ki67 were purchased from Proteintech. Antibodies for iNOS (ab129372) were obtained from Abcam while those for E-cadherin, Vimentin, β-catenin, N-cadherin, p-CDK1, p-Rb, P21, p-IkB α, IKK α/β, p-IKK α/β were purchased from Cell Signaling Technology.

### Radiation for NSCLC cell lines

Flasks (75 cm^2^) with 60% confluent cells were laid with a 1 cm thick compensation above and 50 cm source surface distance (SSD) below, and the field size was 30×30 cm^2^. A549 and H1299 cells were irradiated with X-RAD320ix (Precision X-Ray, North Branford, CT, USA) Following the final radiation, the cells were maintained according to the above culture method in a humidified incubator with 5% CO_2_ at 37° C.

### Colony formation assay

Radioresistance was measured by a clonogenic survival assay. Briefly, 1,000 cells were seeded in 6 cm dishes for 24 hours, and then exposed to a range of radiation doses (2–10 Gy) The medium was replaced regularly and all cultures were incubated for 14 days until the colonies were large enough to be clearly discerned. The positive colonies were defined as groups of >50 cells. The cells were washed with phosphate buffered saline (PBS), fixed with methanol:glacial:acetic (1:1:8) for 10 minutes, and stained with 0.1% crystal violet for 30 minutes. The colonies with more than 50 cells were counted under an optical microscope.

### Cell viability assay

Cell viability was measured by MTT assay. Briefly, the cells were seeded in 96-well plates (2,000 cells/well) and treated the next day with β-elemene, radiation, or the combination of both. MTT was added to the cells 48 hours after treatment, and three hours later, absorbance was measured at 490 nm wavelength. Data were presented as the mean±SD of three independent experiments.

### Wound-healing and transwell migration assay

Wound-healing assay was carried out to measure cell migration. Briefly, cells were plated in 6-well plates, grown to ~70-80% confluence and subjected to different treatments. Cell monolayers were scratched using sterile tips, and the wound gaps were photographed using a Leica DM 14000B microscope. The mean width of each scratch was measured using Image-Pro Plus 5.1 software.

For the transwell migration assay, 4×10^4^ cells were seeded in the upper chambers coated with Matrigel (BD, Biosciences) After administering different treatments, invasive cells on the lower membrane surface were fixed and stained with 0.1% crystal violet. Cells on the underside of the filter were examined by light microscopy and counted in high-power fields.

### Sphere formation

Cells were subjected to different treatments and then trypsinized. The dissociated cells were seeded in an ultra-low attachment plate (Corning, Corning, NY) and suspended in serum-free DMEM/F12 with 1×B27 (Life Technologies), 20 ng/mL epidermal growth factor and 10 ng/mL basic fibroblast growth factor (both from BD biosciences, Bedford, MA) The medium containing growth factors was replaced every three days. After 14 days of culture, spheres (>25 cells) were counted using a light microscope.

### 3D invasion

Spheroid invasion assays were carried out by 3D spheroid BME cell invasion assay (Trevigen catalog no. 3500-096-K) In brief, the cells were trypsinized, pelleted, and resuspended in 50 μl of complete media + 1X Spheroid Formation ECM, and then cultured for three days at 37 ^°^C/5% CO2. After that, spheroids were transferred into the invasion matrix and imaged after a six day incubation.

### Immunofluorescent staining

Cells were cultured on coverslips in 6-well plates and subjected to different treatments. Afterward, the cells were fixed with 4% paraformaldehyde for 30 minutes, permeabilized with 0.2% Triton X-100 in PBS for 5 minutes, and blocked with blocking buffer (10% BSA) for one hour. Then, the cells were incubated with the primary antibody overnight at 4 ^°^C. After washing with PBS, the cells were incubated with the fluorescein isothiocyanate- and rhodamine-conjugated secondary antibodies and DAPI at room temperature. The images were taken with a Leica microscope and processed with Image-Pro Plus 5.1 software.

### Western blotting analysis

Protein levels were determined by Western blotting analysis. Briefly, Proteins from cell and tissue lysates were separated in 10% SDS-PAGE and electrophoretically transferred to polyvinylidene fluoride (PVDF) membranes. Immunoreactive protein bands were detected by enhanced chemiluminescence. All the original data about all Western blots was shown in the Supplementary Figure 1–4.

### Comet assay

Cells were harvested 48 h after being subjected to different treatments. Then, 1.5 × 10^4^ cells from each sample were subjected to the neutral comet assay, as described [18]. Following electrophoresis, the cells were stained with ethidium bromide and visualized using a fluorescence microscope (Leica DMI4000B) We analyzed 200 individual images from each group using Comet Assay Software Pect (CaspLab) Tail moment was defined and served as a quantitative measure of DNA damage.

### RT-PCR

Total RNA was prepared from cultured cells using Trizol Reagent (TaKaRa Bio.) following the procedures suggested by the manufacturer. cDNA synthesis was performed using PrimeScriptTM RT-PCR Kit (TaKaRa) The PCR primers corresponding to Prx-1 (F: ATGTCTTCAGGAAATGCTAAAAT, R: TCACTTCTGCTTGGAGAAATATTC), iNOS (F: TCCAAGGTATCCTGGAGCGA, R: CAGGGACGGGAACTCCTCTA) and GAPDH (F: AATCCCATCACCTCTTCC, R: CATCACGCCACAGTTTCC) functional gene sequences were synthesized by TaKaRa. The PCR products were visualized under ultraviolet light and the band density was measured for quantitative analysis.

### Cell cycle analysis

At 48 hours after treatment, cells were trypsinized, washed with PBS, resuspended in chilled methanol, and kept overnight at 4° C. Cells were then collected, resuspended in 500 μL buffer containing 480 μL PBS, 5 μL RNase, 5 μL PI and 10 μL Triton X- 100, and incubated at 37° C for 30 minutes. After centrifugation, cells were resuspended in 500 μL PBS and filtered. Cell cycle analysis was performed using FACS Calibur™ Flow Cytometer (BD Biosciences, San Jose, CA, USA) The experiments were repeated three times with quadruplicate samples from each treatment.

### Nuclear protein extraction

The cells were lysed in 250 μL cytoplasmic lysis buffer (10 mmol/L Hepes, pH 7.9, 10 mmol/L KCl, 1.5 mmol/L MgCl_2_, 0.5% NP- 40, 300 mmol/L Sucrose) with multiple protease inhibitors (1 mmol/L Na_3_VO_4_, 10 mmol/L NaF, 2.5 mmol/L β- glycerophosphate, 0.1 mmol/L PMSF, 1 g/mL leupeptin, and 0.5 mmol/L dithiothreitol) on ice for 15 minutes. The mixture was vortexed and centrifuged at 12,000*×g* for 10 minutes at 4° C. The supernatant was transferred to a new tube and stored at −80° C. The pellet was resuspended with 70-100 μL nuclear lysis buffer (20 mmol/L Hepes, pH 7.9, 420 mmol/L NaCl, 1.5 mmol/L MgCl_2_, 0.1 mmol/L EDTA, 2.5% glycerol) with multiple protease inhibitors and kept on ice for 30 minutes. Nuclear proteins were extracted by centrifugation at 14,000×*g* for 30 minutes at 40° C. The supernatant was the nuclear extract, and protein concentration was determined by BCA assay.

### *In vivo* tumor model and tissue processing

Female nude mice (4-5 weeks old) were purchased and maintained in SPF laboratory animal central. All animal maintenance and experiment procedures were carried out in accordance with the National Institute of Health Guide for the Care and Use of Laboratory Animals, and approved by Animal Care and Ethics Committee of Dalian Medical University. A549 cells (5×10^6^ in 100 μL PBS) were injected subcutaneously into the left flank of each mouse. When the formed tumor reached 50 mm^3^ after cell inoculation, mice were randomly divided into four groups. Elemene injections were purchased from The Second Affiliated Hospital of Dalian Medical University. Elemene injections were administered via intraperitoneal injection and dosed at 50 mg/kg/day. Radiation was delivered with X-RAD320ix (Precision X-Ray, North Branford, CT, USA) at a single dose of 6 Gy. For combined treatment, radiation was delivered 1 hour after β-elemene was injected. PBS was injected as the control. For limited-dilution *in vivo* experiments, different numbers of cells (1×10^6^, 1×10^5^, 1×10^4^ and 1×10^3^ in 100 μL PBS) were injected subcutaneously into the left flank of each mouse. Tumor volume and body weight were measured every two days. Tumor volume was calculated as V = 1/2 (width^2^ × length) After two weeks, mice were humanely sacrificed by euthanasia. A portion of the tumors were fixed with 10% formalin for immunohistochemical staining, while the remainder of the tumors were used to prepare tumor tissue lysates for Western blot analysis.

### Histology and immunohistochemistry (IHC)

Tumors were fixed in formalin overnight before paraffin embedding. Small tissues were embedded in paraffin for sectioning, incised to 6 μ m thick, and stained with hematoxylin and eosin (H&E) Immunohistochemistry (IHC) was performed using the DAB Kit (Origene, China). All of our microscopy IF pictures were photographed by Leica DM 253 14000B. Larger area for microscopy IF assays was shown in the Supplementary Figure 5.

### Statistical analysis

All the experiments were performed at least three times. Means and standard deviations were calculated from at least three measurements. GraphPad Prism software was used for all statistical analysis. Analysis of variance and Student’s *t*-test were used to compare the values of the test and control samples. Statistical significance was indicated by *P<0.05, **P<0.01, and ***P<0.001.

## Supplementary Material

Supplementary Figures
