# Supplementary material for: β-Elemene enhances radiosensitivity in non-small-cell lung cancer by inhibiting epithelial–mesenchymal transition and cancer stem cell traits via Prx-1/NF-kB/iNOS signaling pathway
Source: Aging (Albany NY). 2020 Dec 9;13(2):2575–92. doi: 10.18632/aging.202291 (PMC7880315; doi:10.18632/aging.202291)
Supplement: Supplementary Figures [file aging-13-202291-s001.pdf]

SUPPLEMENTARY FIGURES

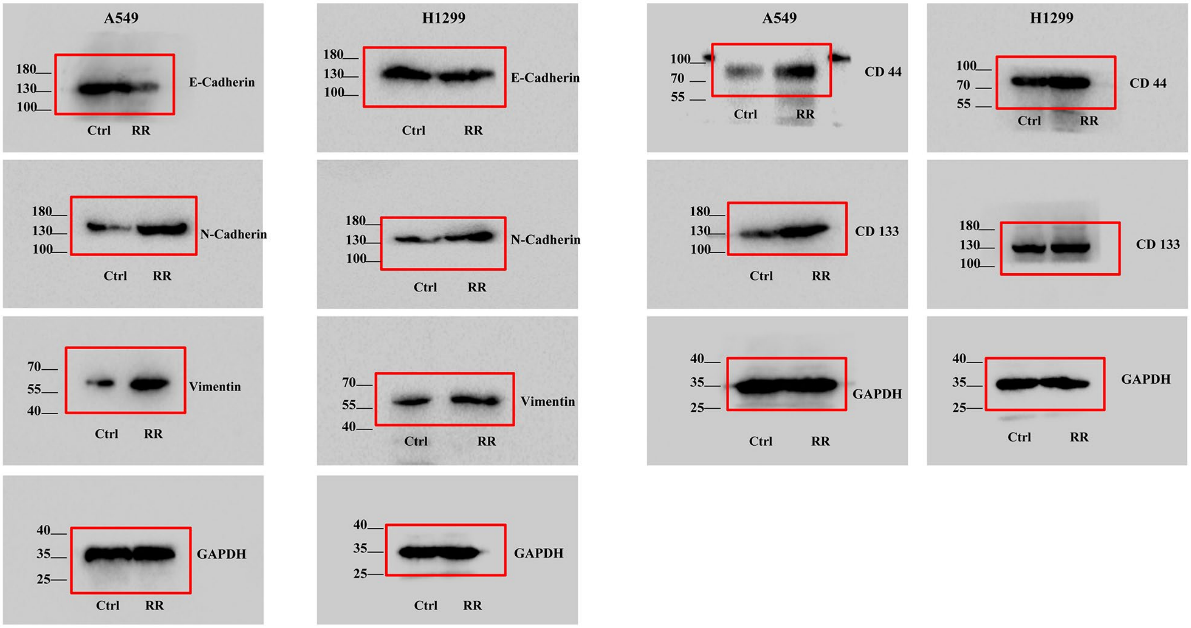

Supplementary Figure 1. The original data of Western blots for Figure 1E, 2C.

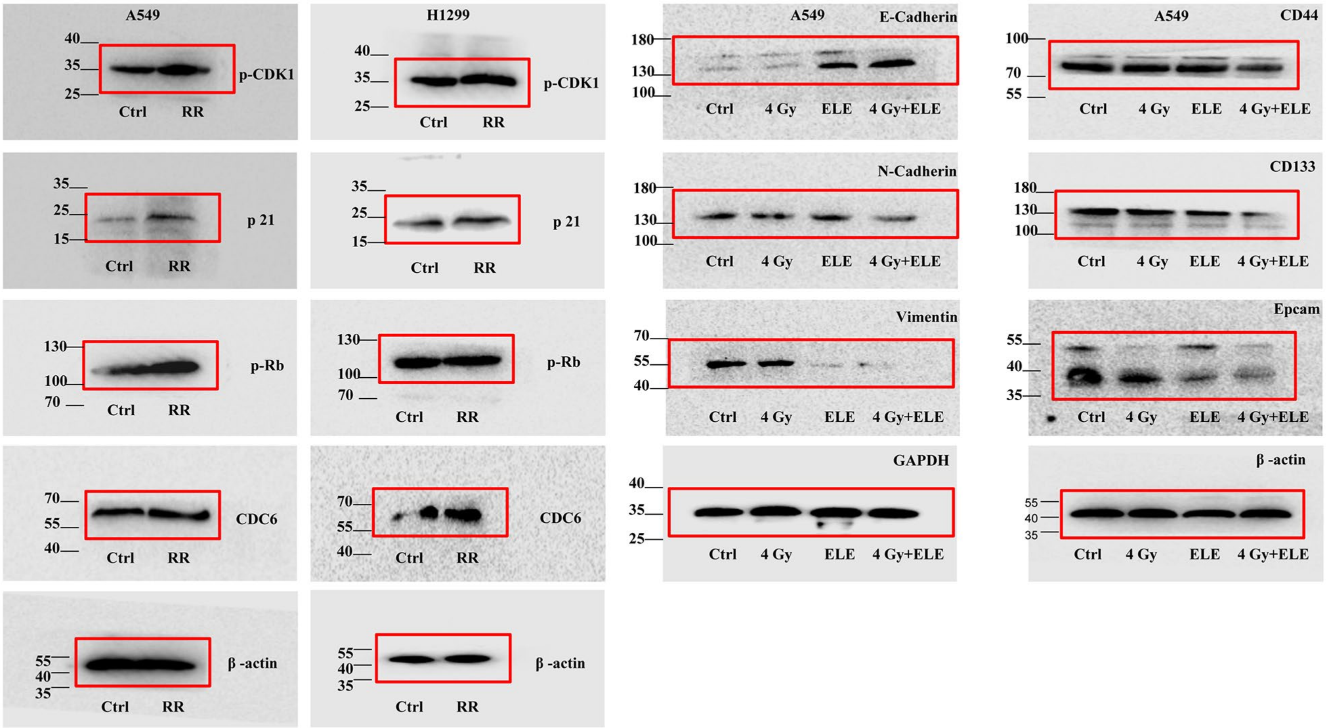

Supplementary Figure 2. The original data of Western blots for Figure 3B, 4D, 4H.

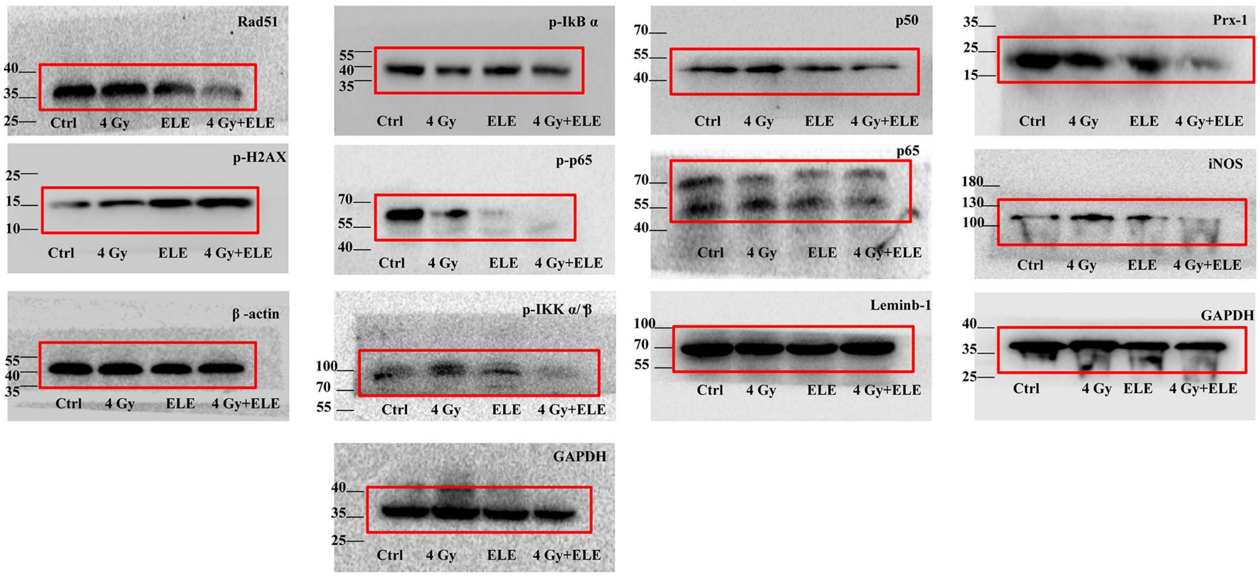

Supplementary Figure 3. The original data of Western blots for Figure 5E, 6A, 6B, 6D.

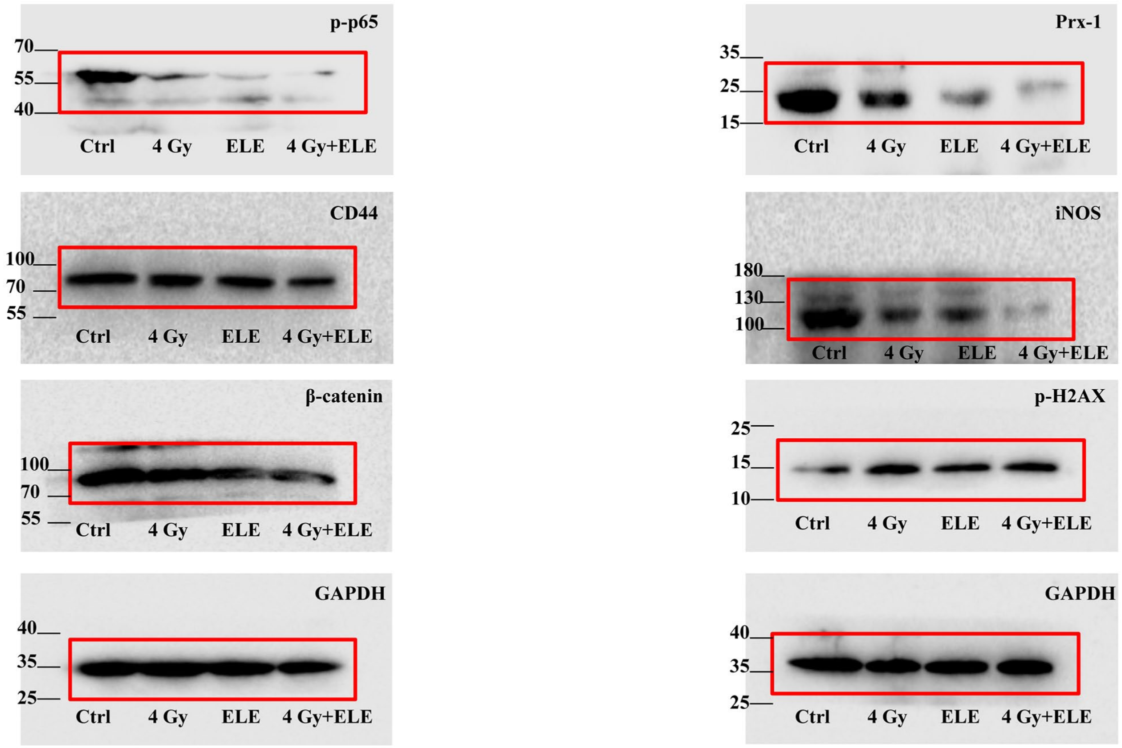

Supplementary Figure 4. The original data of Western blots for Figure 7F.

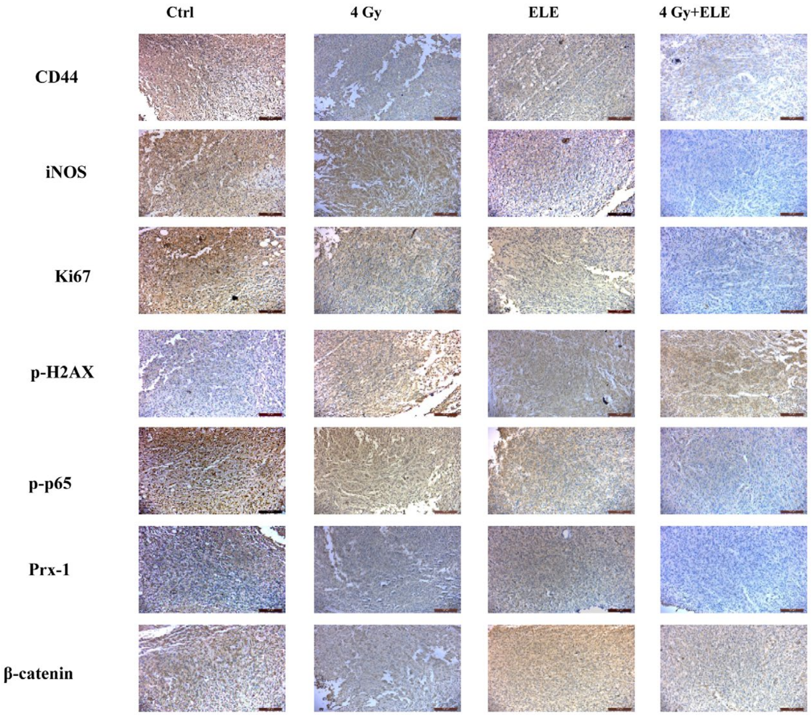

Supplementary Figure 5. Larger area for microscopy IF assays in the Figure 7E.
